# Long‐Term Renal Function and Clinical Outcomes of Sacubitril/Valsartan in HFrEF and Stage 3–4 Chronic Kidney Disease: A 24‐Month Single‐Centre Cohort

**DOI:** 10.1002/clc.70473

**Published:** 2026-09-23

**Authors:** Fatih Akkaya, Onur Osman Şeker, Ercan Türkmen, Seçkin Dereli

**Affiliations:** ^1^ Department of Cardiology Ordu University Faculty of Medicine Ordu Turkey; ^2^ Department of Cardiology Samsun Education and Research Hospital Samsun Turkey; ^3^ Department of Nephrology Ondokuz Mayis University Faculty of Medicine Samsun Turkey

**Keywords:** angiotensin receptor–neprilysin inhibitor, chronic kidney disease, heart failure with reduced ejection fraction, real‐world study, renal outcomes, sacubitril/valsartan

## Abstract

**Background:**

Stage 3–4 chronic kidney disease (CKD) is common in heart failure with reduced ejection fraction (HFrEF) but remains underrepresented in angiotensin receptor–neprilysin inhibitor (ARNI) trials, fostering reluctance to initiate sacubitril/valsartan (S/V).

**Hypothesis:**

We hypothesized that, over 24 months, S/V would be associated with stable renal function and acceptable clinical outcomes; the study was designed to be descriptive rather than comparative.

**Methods:**

We retrospectively analyzed 360 adults with HFrEF (LVEF < 40%) and predialysis stage 3–4 CKD who initiated S/V (2017–2023) and were followed for up to 24 months; decedents were analyzed as events, not excluded. The primary renal composite (sustained ≥ 30% eGFR decline, chronic dialysis, or end‐stage kidney disease) was analyzed with competing‐risk methods (Aalen–Johansen and Fine–Gray, with death competing); mortality with Cox regression; longitudinal eGFR with mixed‐effects; and sparse‐event models with Firth penalization.

**Results:**

Among survivors, mean eGFR was stable (mixed‐model slope non‐significant), with no creatinine rise; NT‐proBNP and CRP fell (both *p* < 0.001) and NYHA class improved. The renal composite occurred in 12/360 (3.3%); all‐cause and cardiovascular mortality were 6.1% and 3.9%. Lower baseline eGFR was associated with the renal composite. Analyzes of maintenance dose and concomitant SGLT2‐inhibitor use were confounded by treatment tolerance and calendar era and were hypothesis‐generating only.

**Conclusions:**

In this single‐arm cohort of HFrEF with stage 3–4 CKD, S/V was generally well tolerated, with stable renal function and low event rates over 24 months. These descriptive, hypothesis‐generating findings cannot establish comparative benefit and require confirmation in controlled studies.

## Practitioner Points

- In a real‐world cohort of HFrEF with stage 3–4 CKD, 24‐month sacubitril/valsartan therapy was associated with stable renal function and low rates of severe renal events and hyperkalaemia.
- Because the cohort is single‐arm and uncontrolled, these descriptive findings are hypothesis‐generating and do not establish comparative renal outcomes and tolerability or support specific up‐titration targets.

## Graphical abstract

In this 24‐month, single‐centre cohort of 360 patients with HFrEF and stage 3–4 chronic kidney disease, sacubitril/valsartan was well tolerated, with stable renal function, improved symptoms, and low rates of renal events and mortality. These descriptive findings are hypothesis‐generating and warrant confirmation in controlled studies.

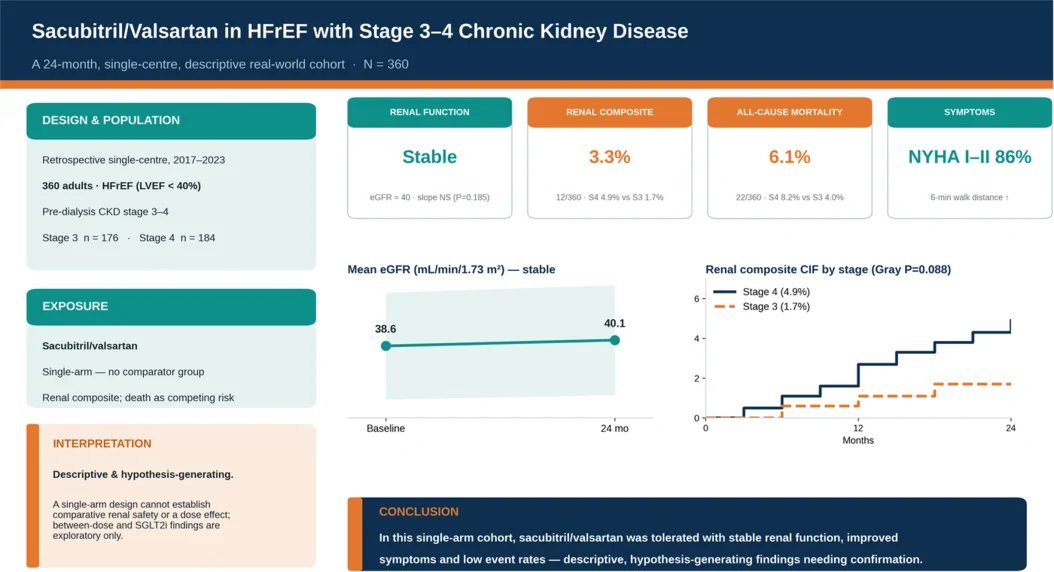

## 1 Introduction

Chronic kidney disease (CKD) and heart failure with reduced ejection fraction (HFrEF) frequently coexist and markedly increase morbidity and mortality [1]. About one third of patients with HFrEF have concomitant CKD, and predialysis stage 3 and 4 CKD is particularly linked to higher hospitalization rates, impaired quality of life and excess all cause and cardiovascular mortality [2]. Yet, despite this high‐risk profile, data to guide renoprotective strategies and optimal medical therapy in HFrEF with stage 3 and 4 CKD remain scarce.

Neurohormonal activation is central to the pathophysiology of both HFrEF and CKD. Increased activity of the renin angiotensin aldosterone system and the sympathetic nervous system drives glomerular hyperfiltration, proteinuria, progressive nephron loss and adverse cardiac remodeling [2]. ACE inhibitors and angiotensin receptor blockers improve cardiac and renal outcomes and are standard therapy, but in older, multimorbid patients with impaired kidney function their initiation and up titration are often limited by hypotension, rises in creatinine and hyperkalaemia [3].

Sacubitril/valsartan, the first angiotensin receptor neprilysin inhibitor (ARNI), reduced cardiovascular mortality and heart failure hospitalizations versus enalapril in the PARADIGM HF trial, and later analyzes suggested a more favorable renal profile than ACEI or ARB, with slower eGFR decline and less progression of albuminuria [4]. However, patients with advanced predialysis CKD were under represented or excluded from pivotal trials, and long‐term data in HFrEF with established stage 3 and 4 CKD, especially under contemporary therapy including SGLT2 inhibitors, remain scarce. Consequently, treatment decisions in this population still depend largely on extrapolation from subgroup and short term observational data, and important uncertainties persist regarding ARNI safety, dosing and overall cardiorenal effects [5].

In this real‐world study, we examined patients with HFrEF and predialysis CKD in stage 3 and 4 treated with sacubitril/valsartan and followed for 24‐months. We aimed to describe changes in renal function (eGFR and serum creatinine), a predefined renal composite endpoint (sustained eGFR decline of at least 30 percent, chronic dialysis or progression to end‐stage kidney disease), and all cause and cardiovascular mortality, together with overall tolerability and adverse events. We also sought to identify simple clinical predictors of renal composite events and mortality, focusing on baseline eGFR and achievement of target sacubitril/valsartan dose. By concentrating on HFrEF with stage 3 and 4 CKD under guideline directed therapy, this study addresses an important evidence gap and may support more informed ARNI use in this high‐risk cardiorenal population.

## 2 Methods

### 2.1 Study Design and Population

This was a retrospective, observational, single‐centre cohort study conducted at a tertiary cardiology referral hospital. Using the electronic hospital information system and individual medical records, we identified all consecutive patients in whom sacubitril/valsartan (SV) therapy was initiated between January 2017 and December 2023 as part of guideline‐directed medical therapy for chronic heart failure with reduced ejection fraction (HFrEF).

**Figure 1:**
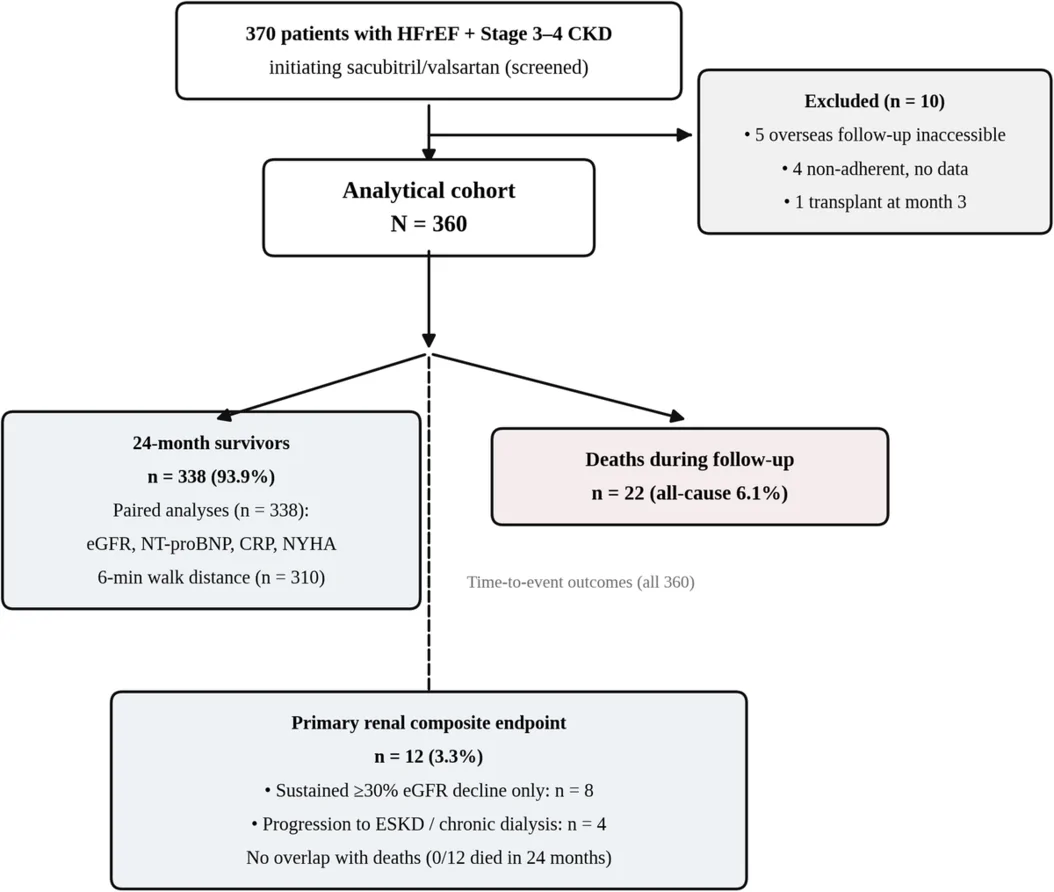
Study flow diagram and analytical cohort. Of 370 consecutive patients with HFrEF and Stage 3–4 CKD initiating sacubitril/valsartan (S/V), 10 were excluded (5 inaccessible overseas follow‐up, 4 non‐adherent without follow‐up data, 1 heart transplantation at month 3), leaving 360 analyzed. At 24 months, 338 survived and contributed to paired longitudinal analyzes (eGFR, NT‐proBNP, CRP and NYHA *n* = 338; 6‐min walk distance *n* = 310); 22 died (all‐cause mortality 6.1%). Twelve patients (3.3%) met the primary renal composite (sustained ≥ 30% eGFR decline [*n* = 8] or ESKD/chronic dialysis [*n* = 4]). No patient with a renal composite event died within 24 months, so the renal (*n* = 12) and mortality (*n* = 22) branches are mutually exclusive. eGFR, estimated glomerular filtration rate; ESKD, end‐stage kidney disease.

Of 370 patients with HFrEF and stage 3–4 CKD initiated on SV during this period, 10 (2.7%) were excluded because 24‐month follow‐up data could not be retrieved (5 dual‐citizen patients residing part of the year abroad; 4 with no regular follow‐up, medication use, or hospital contact; and 1 who underwent heart transplantation at month 3 with subsequent discontinuation of heart‐failure pharmacotherapy), leaving an analytic cohort of 360 (Figure 1). Follow‐up data were obtained through the national e‐Nabız electronic health record system; in‐country loss to follow‐up was therefore negligible. The analytic cohort comprised all 360 patients, of whom 22 died during follow‐up and were retained in every time‐to‐event analysis (analyzed to the date of death); paired baseline‐versus‐24‐month comparisons were restricted to the 338 survivors with paired data, whereas mixed‐effects models used all available measurements from the full cohort, including pre‐death measurements of decedents. CKD stage (3 vs 4) was assigned at baseline (T0) and used as a fixed grouping variable; patients were not reclassified during follow‐up.

#### 2.1.1 Patients Were Eligible for Inclusion If They Fulfilled the Following Criteria at the Time of SV Initiation

- Clinical diagnosis of chronic heart failure with reduced ejection fraction;
- Left ventricular ejection fraction (LVEF) < 40% on transthoracic echocardiography;
- New York Heart Association (NYHA) functional class II–IV;
- Chronic kidney disease (CKD) stage 3 or stage 4 according to KDIGO criteria, defined by an estimated glomerular filtration rate (eGFR) 15–59 mL/min/1.73 m^2^ (CKD‐EPI equation);
- No previous exposure to sacubitril/valsartan (ARNI‐naïve).

Prior treatment with angiotensin‐converting enzyme inhibitors (ACEIs) or angiotensin II receptor blockers (ARBs) was not an exclusion criterion; both patients previously treated with ACEI/ARB and RAAS‐naïve patients were eligible and were included.

#### 2.1.2 Exclusion Criteria Were

- Acute coronary syndrome at the index presentation or within 30 days before SV initiation;
- Acute kidney injury at baseline;
- Major surgery or significant trauma within the preceding 3 months;
- Active malignancy;
- CKD stage 5 (eGFR < 15 mL/min/1.73 m^2^) or chronic maintenance dialysis;
- Pregnancy or breastfeeding;
- History of angioedema;
- Missing key baseline data required for eligibility assessment, and complete loss of follow‐up (no retrievable post‐baseline data), were exclusion criteria. Availability of 24‐month measurements was not an exclusion criterion for the full cohort; it defined only the longitudinal sub‐cohort used for the paired baseline‐versus‐24‐month comparisons (see Statistical analysis).

### 2.2 Sacubitril/Valsartan Treatment and Titration

SV was prescribed at the discretion of the treating cardiologist in accordance with contemporary heart failure guidelines. In patients previously receiving an ACEI, a 36‐h washout period was applied whenever feasible before starting SV. The initial SV dose was selected on the basis of seated blood pressure, age and frailty, hepatic and renal function, and the prior ACEI/ARB dose, and was 24/26, 49/51 or 97/103 mg twice daily.

Dose up‐titration was assessed during routine clinical visits, generally every 2–8 weeks. Escalation towards the target dose of 97/103 mg twice daily was attempted in the absence of intolerance, aiming for:

- seated systolic blood pressure (SBP) ≥ 100 mmHg without orthostatic symptoms,
- serum potassium ≤ 5.2 mmol/L,
- no ≥ 30% decline in eGFR from the individual's baseline value and no episode of acute kidney injury,
- no interim haemodynamic or clinical decompensation.

Most pivotal randomized trials and real‐world cohorts of HFrEF treated with SV have enrolled patients with eGFR ≥ 30 mL/min/1.73 m^2^, and this range therefore represents the main evidence base for its use. Importantly, neither the ESC heart failure guidelines nor the AHA/ACC/HFSA guidelines formally contraindicate RAAS blockade or ARNI therapy in patients with more advanced CKD; instead, they recommend close monitoring of renal function and potassium, individualized dosing, and joint decision‐making with nephrology in patients with eGFR < 30 mL/min/1.73 m^2^ [6, 7]. In line with this guidance, patients with baseline eGFR 15–29 mL/min/1.73 m^2^ (CKD stage 4) were considered for up‐titration on an individual basis, provided that renal function remained stable (no ≥ 30% decline from baseline) and all of the above safety criteria were satisfied.

Conversely, symptomatic hypotension ora systolic blood pressure < 95 mmHg (and invariably < 90 mmHg) potassium ≥ 5.5 mmol/L, a ≥ 30% decline in eGFR, clinically relevant brady‐ or tachyarrhythmias, or other significant adverse effects prompted SV dose reduction, temporary interruption or permanent discontinuation, at the discretion of the physician. Management of SV dosing was dynamic throughout follow‐up, balancing the goal of achieving the highest tolerated dose against safety considerations.

The maintenance dose was defined as the highest sacubitril/valsartan dose that each patient could reach and sustain long‐term while keeping blood pressure, kidney function and serum potassium within an acceptable range. Transient dose reduction or interruption owing to short‐lived hypotension, changes in creatinine or potassium, or intercurrent illness did not alter the assigned maintenance dose; when a patient durably tolerated only a lower dose, that was taken as the maintenance dose. Dose optimization was undertaken within patient‐tailored quadruple guideline‐directed therapy.

### 2.3 Concomitant Therapies and Guideline Context

Background heart failure therapy (beta‐blockers, mineralocorticoid receptor antagonists, loop diuretics, ivabradine, ACEI/ARB before switch, and device therapy) was optimized as far as possible according to the ESC heart failure guidelines in force at the time of each patient's treatment decision [6].

The inclusion period (2017–2023) spans a phase of substantial evolution in guideline‐directed therapy. SGLT‐2 inhibitors were incorporated as a core component of HFrEF management in the 2021 ESC heart failure guideline update and are strongly recommended in recent KDIGO CKD guidelines for patients with diabetic and non‐diabetic CKD [6, 8]. As a result:

- patients who initiated SV before 2021 and were not already on an SGLT‐2 inhibitor generally did not receive these agents during follow‐up;
- after 2021, prescription of SGLT‐2 inhibitors increased markedly in eligible HFrEF patients with stage 3–4 CKD.

This temporal change accounts for the non‐uniform distribution of SGLT‐2 inhibitor use in the cohort and was considered when interpreting subgroup analyzes by SGLT‐2 status.

Because SGLT2 inhibitors became a Class I recommendation only with the 2021 ESC guideline, their use rose during 2017–2023; baseline user‐versus‐non‐user status is therefore confounded by calendar period. SGLT2 inhibitor exposure was modeled as a time‐varying covariate (date of initiation) with adjustment for calendar era (before vs after 2021) in time‐dependent Cox, and reported as an adjusted hazard ratio rather than an unstratified Kaplan–Meier comparison. These analyzes are exploratory and do not support causal inference regarding combined ARNI–SGLT2 inhibitor therapy.

#### 2.3.1 Clinical and Laboratory Assessments

Demographic characteristics (age, sex, smoking status), heart failure duration, aetiology (ischaemic vs non‐ischaemic), comorbidities (hypertension, diabetes mellitus, atrial fibrillation, chronic obstructive pulmonary disease, dyslipidaemia, prior valve surgery) and device therapies (cardiac resynchronization therapy [CRT] and/or implantable cardioverter‐defibrillator [ICD]) were extracted from medical records.

Physical examination variables included systolic and diastolic blood pressure, heart rate, body weight and body mass index (BMI). Echocardiographic parameters (left ventricular end‐diastolic diameter and LVEF) were measured using standard techniques.

Laboratory data comprised serum sodium, potassium, hemoglobin, albumin, total cholesterol, serum creatinine and C‐reactive protein (CRP). Estimated GFR was calculated using the CKD‐EPI equation, and CKD stage (3 vs 4) was assigned according to KDIGO definitions. N‐terminal pro‐B‐type natriuretic peptide (NT‐proBNP) levels were recorded at baseline and at follow‐up; owing to their skewed distribution, NT‐proBNP values were log‐transformed (log NT‐proBNP) for regression analyzes.

Following the initial review, records were re‐examined in detail. Baseline albuminuria was recorded, where available (n reported), as UACR from nephrology and internal‐medicine notes and categorized by KDIGO criteria (A1 < 30; A2 30–300; A3 > 300 mg/g). Loop diuretics and the four components of guideline‐directed therapy, with timing of initiation and discontinuation, were verified not only from clinical documentation but also against the national Medula prescription/pharmacy‐dispensing system, providing objective, dispensing‐based confirmation of drug exposure, initiation dates and continuity of use.

Functional status was assessed using the NYHA classification at baseline and at the 24‐month visit. For pre–post comparisons and visualization of transitions (Sankey diagram), NYHA class at these two time points was used.

At every visit patients underwent structured symptom review, physical examination and volume‐status assessment; a 6‐min walk test (6MWT) was obtained at baseline in 328 patients (baseline data available); the paired baseline–follow‐up analysis included 310 patients and repeated in clinically deteriorating patients and routinely about 6 months after the maintenance dose was achieved (the follow‐up interval was not uniform). To avoid survivor bias, NYHA class was additionally analyzed as an ordinal outcome across the full cohort (*n* = 360) in a responder analysis in which patients who died were assigned the worst category (death ranked below NYHA IV); this conservative approach penalizes rather than favors the intervention.

Baseline values were defined as those obtained closest to SV initiation, and follow‐up values as those obtained at, or as close as possible to, the 24‐month time point.

### 2.4 Endpoints

The primary endpoint was a renal composite outcome occurring within 24‐months after SV initiation, defined as the occurrence of at least one of the following:

1. A sustained ≥ 30% decline in eGFR from baseline, confirmed by at least two measurements ≥ 3 months apart;

Only chronic, sustained declines confirmed by at least two measurements ≥ 3 months apart were counted; transient or reversible (recovered) worsening was not counted as an event.

1. Initiation of chronic dialysis (hemodialysis or peritoneal dialysis);
2. Progression to end‐stage kidney disease, defined as a sustained eGFR < 15 mL/min/1.73 m^2^.

Secondary endpoints were:

1. All‐cause mortality: death from any cause within 24‐months;
2. Cardiovascular mortality: death due to worsening heart failure, sudden cardiac death, myocardial infarction or stroke;
3. Adverse events during 24‐month follow‐up, specifically:
  ◦ clinically relevant hyperkalaemia documented by laboratory testing and/or requiring treatment modification; Hyperkalaemia was defined as a serum potassium ≥ 5.5 mmol/L.
  ◦ symptomatic hypotension attributed to SV; Symptomatic hypotension was defined as systolic blood pressure < 100 mmHg accompanied by temporally related symptoms (dizziness, fatigue, or light‐headedness, particularly on standing).
  ◦ temporary interruption or permanent discontinuation of SV;
  ◦ sustained ≥ 30% eGFR decline and initiation of chronic dialysis.

Renal and mortality outcomes were analyzed separately: the renal composite with death treated as a competing risk, and all‐cause and cardiovascular mortality by Kaplan–Meier and Cox. No combined mortality–renal composite endpoint was used.

### 2.5 Statistical Analysis

Continuous variables are presented as mean ± standard deviation (SD) for approximately normally distributed data or median (interquartile range) for skewed distributions; categorical variables are expressed as counts and percentages. Normality was assessed using the Kolmogorov–Smirnov test and visual inspection of histograms.

Paired comparisons between baseline and 24‐month follow‐up were performed using the paired‐samples *t*‐test for normally distributed variables or the Wilcoxon signed‐rank test for non‐normal variables. Changes in categorical variables, such as NYHA class distribution, were evaluated using the McNemar test or related procedures, as appropriate.

The primary renal composite was analyzed as a time‐to‐event outcome with death treated as a competing risk (Fine–Gray subdistribution and cause‐specific Cox); components (sustained ≥ 30% eGFR decline, chronic dialysis, end‐stage kidney disease) are reported separately, and each patient is counted once at the first qualifying event. All‐cause mortality was analyzed by Kaplan–Meier and Cox, and cardiovascular mortality by Aalen–Johansen cumulative incidence with non‐cardiovascular death as a competing event (Gray's test); no combined mortality–renal composite was used, and renal and mortality outcomes are shown in separate figures. Stage‐stratified analyzes used the full cohort from time zero. Because dose is titrated after initiation, dose‐stratified analyzes used a 3‐month landmark and classified patients by the dose prescribed at the 3‐month landmark (median time to dose optimization 2.2 months, IQR: 1.4–3.1); events before the landmark are reported and excluded from the landmark population, with time‐dependent Cox as a sensitivity analysis. Starting dose, 3‐month landmark dose and 24‐month long‐term maintenance dose are reported separately. Given the limited number of events (22 deaths; 12 renal composite events), multivariable models were pre‐specified and simplified and estimated with Firth penalization, with an internal bootstrap stability assessment (1,000 resamples; coefficient sign‐consistency and percentile confidence intervals); model estimates are reported as exploratory associations rather than as independent predictors [9, 10]. Longitudinal eGFR was analyzed with a linear mixed‐effects model using all serial measurements (fixed effects: time modeled linearly, CKD stage and a time × stage interaction; random intercept and random slope for time per patient; unstructured covariance), from which the mean annual eGFR slope with 95% confidence interval is reported; adjusted contrasts were adjusted for age, sex, baseline eGFR and diabetes, and model assumptions were checked by inspection of residuals. Renal and safety outcomes are additionally reported separately for CKD stage 3 and stage 4 [11, 12].

Univariable Fine–Gray models were used to estimate subdistribution hazard ratios for the renal composite, with all‐cause death treated as a competing event. Given the limited number of renal events, an exploratory two‐predictor Firth‐penalized cause‐specific Cox model including baseline eGFR and log_2_‐transformed UACR was fitted as a multivariable sensitivity analysis. Results are reported as exploratory associations rather than independent predictors.

For all‐cause mortality (22 events), a single pre‐specified Firth‐penalized Cox model with four a‐priori covariates (age, baseline NYHA class III–IV, log NT‐proBNP and baseline eGFR) was fitted; sacubitril/valsartan dose was deliberately excluded from the baseline model to avoid immortal‐time bias and was examined separately by 3‐month landmark analysis. Estimates are reported as exploratory hazard ratios with profile‐likelihood 95% confidence intervals; because events per variable were low (5.5) and all P‐values were borderline (0.018–0.046), these associations should be regarded as hypothesis‐generating.

Time‐to‐event analyzes for each endpoint (the renal composite and all‐cause mortality) analyzed separatelywere conducted using Kaplan–Meier survival curves, and between‐group differences were evaluated with the log‐rank test. Pre‐specified subgroup analyzes were performed according to:

- maintenance SV dose (24/26, 49/51, 97/103 mg twice daily),
- CKD stage (stage 3 vs stage 4),
- SGLT‐2 inhibitor use during follow‐up [yes vs no].

Because SGLT‐2 inhibitor uptake increased after 2021, we also performed a sensitivity analysis stratified by enrollment period (before vs after 2021). Findings were interpreted as exploratory.

All statistical tests were two‐sided, and a *p*‐value < 0.05 was considered statistically significant. Statistical analyzes were carried out using IBM SPSS Statistics, version 28.0 (IBM Corp., Armonk, NY, USA).

The study protocol was approved by the local Institutional Ethics Committee and conducted in accordance with the principles of the Declaration of Helsinki. Owing to the retrospective nature of the study, the requirement for written informed consent was waived.

## 3 Results

### 3.1 Baseline Characteristics

A total of 360 patients with HFrEF and predialysis CKD in stage 3 and 4 who started sacubitril/valsartan and were followed for up to 24 months, including patients who died during follow‐up, were included. Mean age was 63.4 ± 10.8 years, 65 percent were male, HF duration was 6.2 ± 3.5 years and ischaemic aetiology was present in 73 percent. At baseline, mean blood pressure was 119.2 ± 18.4 over 65.8 ± 10.2 mmHg, heart rate 72.9 ± 11.4 beats per minute, body weight 75.9 ± 17 kg and body mass index 26.8 ± 6.2 kg per square metre (Table 1a).

**Table 1a:** Baseline characteristics according to chronic kidney disease stage.

Characteristic	Total (*N* = 360)	CKD Stage 3 (*n* = 176)	CKD Stage 4 (*n* = 184)	*p*
Age (years)	63.4 ± 10.8	62.1 ± 10.5	64.6 ± 11.0	0.032
Male sex, *n* (%)	234 (65.0)	116 (65.9)	118 (64.1)	0.724
Ischemic etiology, *n* (%)	262 (72.8)	126 (71.6)	136 (73.9)	0.621
HF duration (years)	6.2 ± 3.5	6.0 ± 3.4	6.4 ± 3.6	0.284
Diabetes mellitus, *n* (%)	162 (45.0)	69 (39.2)	93 (50.5)	0.031
Hypertension, *n* (%)	248 (68.9)	114 (64.8)	134 (72.8)	0.098
Atrial fibrillation, *n* (%)	112 (31.1)	50 (28.4)	62 (33.7)	0.281
CRT and/or ICD, *n* (%)	108 (30.0)	56 (31.8)	52 (28.3)	0.468
Heart rate (beats/min)	72.9 ± 11.4	73.4 ± 11.2	72.4 ± 11.6	0.402
LVEF (%)	28.3 ± 5.4	28.8 ± 5.2	27.8 ± 5.5	0.078
LVEDD (mm)	59.1 ± 6.5	58.8 ± 6.2	59.4 ± 6.8	0.380
Systolic BP (mmHg)	119.2 ± 18.4	120.6 ± 17.8	117.8 ± 18.9	0.148
Diastolic BP (mmHg)	65.8 ± 10.2	66.4 ± 9.8	65.2 ± 10.6	0.264
eGFR (mL/min/1.73 m^2^)	38.0 ± 13.8	51.2 ± 5.8	25.4 ± 3.6	< 0.001
Serum creatinine (mg/dL)	1.74 ± 0.52	1.36 ± 0.30	2.10 ± 0.46	< 0.001
UACR (mg/g) (*n* = 336)	185 [65–420]	120 [45–260] (*n* = 164)	280 [110–680] (*n* = 172)	< 0.001
Serum albumin (g/dL)	3.8 ± 0.4	3.9 ± 0.4	3.7 ± 0.4	< 0.001
Serum potassium (mmol/L)	4.6 ± 0.5	4.5 ± 0.4	4.7 ± 0.5	< 0.001
Hemoglobin (g/dL)	12.8 ± 1.6	13.2 ± 1.5	12.4 ± 1.6	< 0.001
NT‐proBNP (ng/L)	1420 [850–2840]	1150 [680–2100]	1840 [1120–3450]	< 0.001
CRP (mg/L)	1.7 [0.8–4.2]	1.5 [0.7–3.6]	2.1 [0.9–5.1]	0.004
NYHA III–IV, *n* (%)	236 (65.6)	100 (56.8)	136 (73.9)	< 0.001
6‐min walk distance (m) (*n* = 328)	316 ± 69	339 ± 61 (*n* = 162)	293 ± 69 (*n* = 166)	< 0.001
≥ 1 HF hosp/1 yr, *n* (%)	198 (55.0)	82 (46.6)	116 (63.0)	0.002
HF hosp/pt in 1 yr	1 [0–1]	0 [0–1]	1 [0–2]	< 0.001
Baseline GDMT medications				
ACEi/ARB before switch, *n* (%)	234 (65.0)	122 (69.3)	112 (60.9)	0.094
Beta‐blocker, *n* (%)	302 (83.9)	150 (85.2)	152 (82.6)	0.499
MRA, *n* (%)	216 (60.0)	118 (67.0)	98 (53.3)	0.008
SGLT2 inhibitor, *n* (%)	126 (35.0)	72 (40.9)	54 (29.3)	0.022
Loop diuretic user, *n* (%)	274 (76.1)	124 (70.5)	150 (81.5)	0.014
Loop diuretic dose (mg/d furo eq.)	58 ± 26	48 ± 22	68 ± 27	< 0.001
Four‐pillar GDMT user, *n* (%)	84 (23.3)	52 (29.5)	32 (17.4)	0.007
Baseline S/V starting dose, *n* (%)				0.009
24/26 mg bid	168 (46.7)	68 (38.6)	100 (54.3)	
49/51 mg bid	156 (43.3)	86 (48.9)	70 (38.0)	
97/103 mg bid	36 (10.0)	22 (12.5)	14 (7.6)	

*Note:* Mean ± SD, median [IQR], or *n* (%). Welch *t*‐test/Mann–Whitney U; categorical Pearson chi‐square, Fisher–Freeman–Halton for 2 × 3 dose. Four‐pillar GDMT = ACEi/ARB + beta‐blocker + MRA + SGLT2i before S/V. UACR and 6MWD complete‐case.

Abbreviations: 6MWD, 6‐min walk distance; ACEi, angiotensin‐converting enzyme inhibitor; ARB, angiotensin receptor blocker; bid, twice daily; CKD, chronic kidney disease; CRP, C‐reactive protein; eGFR, estimated glomerular filtration rate; GDMT, guideline‐directed medical therapy; HFrEF, heart failure with reduced ejection fraction; IQR, interquartile range; LVEF, left ventricular ejection fraction; MRA, mineralocorticoid receptor antagonist; NT‐proBNP, *N*‐terminal pro–B‐type natriuretic peptide; NYHA, New York Heart Association; SD, standard deviation; SGLT2i, sodium‐glucose cotransporter‐2 inhibitor; S/V, sacubitril/valsartan; UACR, urine albumin‐to‐creatinine ratio.

**Table 1b:** Baseline characteristics according to 24‐month vital status (survivors vs non‐survivors).

Characteristic	Survivors (*n* = 338)	Non‐Survivors (*n* = 22)	*p*
Age (years)	62.8 ± 10.5	72.5 ± 9.8	< 0.001
Male sex, *n* (%)	220 (65.1)	14 (63.6)	1.000
Ischemic etiology, *n* (%)	245 (72.5)	17 (77.3)	0.805
HF duration (years)	6.1 ± 3.4	7.8 ± 4.1	0.070
Diabetes mellitus, *n* (%)	148 (43.8)	14 (63.6)	0.079
Hypertension, *n* (%)	231 (68.3)	17 (77.3)	0.480
Atrial fibrillation, *n* (%)	103 (30.5)	9 (40.9)	0.344
CRT and/or ICD, *n* (%)	100 (29.6)	8 (36.4)	0.482
Heart rate (beats/min)	72.7 ± 11.2	75.8 ± 13.5	0.303
LVEF (%)	28.6 ± 5.3	24.1 ± 5.1	< 0.001
LVEDD (mm)	59.0 ± 6.4	60.6 ± 7.6	0.348
Systolic BP (mmHg)	119.5 ± 18.2	114.6 ± 21.0	0.301
Diastolic BP (mmHg)	66.0 ± 10.1	62.7 ± 11.4	0.198
eGFR (mL/min/1.73 m^2^)	38.6 ± 13.8	28.8 ± 9.4	< 0.001
Serum creatinine (mg/dL)	1.71 ± 0.52	2.18 ± 0.58	0.001
UACR (mg/g)	170 [60–380] (*n* = 316)	540 [260–1150] (*n* = 20)	< 0.001
Serum albumin (g/dL)	3.8 ± 0.4	3.5 ± 0.4	0.002
Serum potassium (mmol/L)	4.6 ± 0.5	4.7 ± 0.6	0.412
Hemoglobin (g/dL)	12.9 ± 1.5	11.8 ± 1.9	0.018
NT‐proBNP (ng/L)	1350 [810–2650]	3850 [2400–6200]	< 0.001
CRP (mg/L)	1.6 [0.8–3.9]	3.4 [1.8–7.5]	< 0.001
NYHA functional class, *n* (%)			< 0.001
NYHA Class II	122 (36.1)	2 (9.1)	
NYHA Class III	178 (52.7)	8 (36.4)	
NYHA Class IV	38 (11.2)	12 (54.5)	
6‐min walk distance (m)	322 ± 66 (*n* = 310)	208 ± 54 (*n* = 18)	< 0.001
≥ 1 HF hosp/1 yr, *n* (%)	178 (52.7)	20 (90.9)	< 0.001
HF hosp/pt in 1 yr	1 [0–1]	2 [1–2]	< 0.001
CKD Stage 4, *n* (%)	169 (50.0)	15 (68.2)	0.124
Baseline GDMT medications			
ACEi/ARB before switch, *n* (%)	221 (65.4)	13 (59.1)	0.645
Beta‐blocker, *n* (%)	285 (84.3)	17 (77.3)	0.373
MRA, *n* (%)	204 (60.4)	12 (54.5)	0.656
SGLT2 inhibitor, *n* (%)	120 (35.5)	6 (27.3)	0.497
Loop diuretic user, *n* (%)	254 (75.1)	20 (90.9)	0.122
Loop diuretic dose (mg/d furo eq.)	55 ± 24	96 ± 32	< 0.001
Four‐pillar GDMT user, *n* (%)	80 (23.7)	4 (18.2)	0.795
Baseline S/V starting dose, *n* (%)			0.127
24/26 mg bid	153 (45.3)	15 (68.2)	
49/51 mg bid	150 (44.4)	6 (27.3)	
97/103 mg bid	35 (10.4)	1 (4.5)	

*Note:* Mean ± SD, median [IQR], or *n* (%). Welch *t*‐test/Mann–Whitney U; two‐sided Fisher exact, Fisher–Freeman–Halton for 2 × 3 NYHA and dose. Univariate, exploratory; not used for data‐driven covariate selection.

Abbreviations: 6MWD, 6‐min walk distance; CKD, chronic kidney disease; CRP, C‐reactive protein; eGFR, estimated glomerular filtration rate; IQR, interquartile range; NT‐proBNP, N‐terminal pro–B‐type natriuretic peptide; NYHA, New York Heart Association; SD, standard deviation; S/V, sacubitril/valsartan; UACR, urine albumin‐to‐creatinine ratio.

Comorbidity burden was high: atrial fibrillation in 31 percent, hypertension in 69 percent, diabetes in 45 percent, dyslipidaemia in 33 percent, chronic obstructive pulmonary disease in 12 percent, prior valve surgery in 12 percent and cardiac resynchronization therapy and or implantable cardioverter defibrillator in 30 percent. Mean left ventricular ejection fraction was 28.3 ± 5.4 percent, mean eGFR 38.0 ± 13.8 mL/min/1.73 m^2^ and serum creatinine 1.74 ± 0.52 mg/dL; median NT‐proBNP and CRP were 1420 [850–2840] ng/L and 1.7 mg/L. At baseline, 34 percent of patients were in NYHA class II, 52 percent in class III and 14 percent in class IV; 49 percent had CKD in stage 3 and 51 percent in stage 4. Guideline directed therapy was frequent, including ACEI or ARB in 65 percent, beta blocker in 84 percent, mineralocorticoid receptor antagonist in 60 percent, SGLT2 inhibitor in 35 percent, loop diuretic in 76 percent, ivabradine in 22 percent and statin in 21 percent (Table 1a).

### 3.2 Sacubitril/Valsartan Dosing and Concomitant Therapy

During follow‐up, sacubitril/valsartan was maintained at 24 or 26 mg twice daily in 84 patients (23 percent), 49 or 51 mg twice daily in 172 (48 percent) and 97 or 103 mg twice daily in 104 (29 percent), so almost one third reached the target dose while about half remained on an intermediate dose. In parallel, guideline directed therapy was further optimized: mineralocorticoid receptor antagonist use rose from 60 to 72 percent and SGLT2 inhibitor use from 35 to 80 percent, whereas loop diuretic use fell from 76 to 62 percent (all *p* = 0.001), with only minor changes in beta blocker, ivabradine and statin therapy (Table 2). Detailed treatment transitions and starting‐to‐maintenance dose transitions are provided in Tables S1 and S3.

**Table 2:** Paired changes from baseline to 24 months among survivors (up to *n* = 338), with longitudinal mixed‐model estimates (up to *N* = 360).

Parameter	Baseline (T0)	24‐Month FU	Paired change (95% CI)	Paired *p*	LMM effect (up to *N* = 360)	LMM *p*
Renal function & Proteinuria						
eGFR (mL/min/1.73 m^2^)	38.6 ± 13.8	40.1 ± 14.2	+1.5 (+0.5 to +2.5)	0.004	Monthly slope +0.04/mo (−0.02 to +0.10) (*k* = 1984)	0.185
Serum creatinine (mg/dL)	1.71 ± 0.52	1.68 ± 0.50	−0.03 (−0.06 to −0.002)	0.038	Adj 24‐mo contrast −0.02 (−0.07 to +0.03)	0.412
UACR (mg/g) (cc *n* = 316)	170 [60–380]	135 [50–310]	HL −30 (−45 to −15)	< 0.001	Adj GMR 0.81 (0.74–0.89) (*N* = 336, *k* = 1420)	< 0.001
Serum potassium (mmol/L)	4.62 ± 0.48	4.68 ± 0.51	+0.06 (−0.01 to +0.13)	0.114	Adj 24‐mo contrast +0.05 (−0.02 to +0.12)	0.162
Serum sodium (mmol/L)	137.2 ± 5.1	138.1 ± 4.9	+0.9 (−0.1 to +1.9)	0.084	—	—
Hemoglobin (g/dL)	12.9 ± 1.5	13.0 ± 1.4	+0.1 (−0.1 to +0.3)	0.312	—	—
Neurohormonal & Inflammatory						
NT‐proBNP (ng/L)	1350 [810–2650]	360 [155–980]	HL −920 (−1080 to −760)	< 0.001	Adj GMR 0.29 (0.25–0.34) (*k* = 1,984)	< 0.001
CRP (mg/L)	1.6 [0.8–3.9]	1.1 [0.5–2.5]	HL −0.5 (−0.8 to −0.3)	< 0.001	—	—
Functional status & Decongestion						
6‐min walk distance (m) (*n* = 310)	322 ± 66 (*n* = 310)	388 ± 71	+66 (+59 to +73)	< 0.001	—	—
NYHA class, *n* (%) [4 × 4 shift]			Stuart–Maxwell	< 0.001	—	—
NYHA I	0 (0.0)	138 (40.8)				
NYHA II	122 (36.1)	154 (45.6)				
NYHA III	178 (52.7)	46 (13.6)				
NYHA IV	38 (11.2)	0 (0.0)				
Loop diuretic user, *n* (%)	254 (75.1)	208 (61.5)	−13.6 pp	< 0.001	—	—
Loop diuretic dose (mg/d furo eq.)	55 ± 24	36 ± 18	−19 (−22 to −16)	< 0.001	—	—
HF hospitalizations per pt‐year	0.78 ± 0.72	0.21 ± 0.44	−0.57 (−0.66 to −0.48)	< 0.001	—	—
GDMT optimization & S/V Dosing						
MRA user, *n* (%)	204 (60.4)	246 (72.8)	+12.4 pp	< 0.001	—	—
SGLT2 inhibitor user, *n* (%)	120 (35.5)	274 (81.1)	+45.6 pp	< 0.001	—	—
Beta‐blocker user, *n* (%)	285 (84.3)	291 (86.1)	+1.8 pp	0.210	—	—
Four‐pillar GDMT user, *n* (%)	80 (23.7)	242 (71.6)	+47.9 pp	< 0.001	—	—
Maintenance dose [3 × 3 shift], *n* (%)			Stuart–Maxwell	< 0.001	—	—
24/26 mg bid (Low)	153 (45.3)*	78 (23.1)				
49/51 mg bid (Interm.)	150 (44.4)*	160 (47.3)				
97/103 mg bid (Target)	35 (10.4)*	100 (29.6)				

*Note:* Survivors up to *n* = 338 (UACR cc *n* = 316; 6MWD *n* = 310). Paired *t*‐test/Wilcoxon; exact McNemar (binary); Stuart–Maxwell for multi‐category (NYHA and dose). The follow‐up 6‐min walk distance was obtained at a non‐uniform interval (approximately 6 months after dose optimization), not at a fixed 24‐month timepoint.

Abbreviations: 6MWD, 6‐min walk distance; bid, twice daily; CRP, *C*‐reactive protein; eGFR, estimated glomerular filtration rate; HL, Hodges–Lehmann shift; IQR, interquartile range; LMM, linear mixed model; NT‐proBNP, *N*‐terminal pro–B‐type natriuretic peptide; NYHA, New York Heart Association; S/V, sacubitril/valsartan; SD, standard deviation; UACR, urine albumin‐to‐creatinine ratio.

* Baseline starting‐dose distribution. LMM used all measurements while alive at T0, 3, 6, 12, 18, 24 mo (*k* = 1,984; UACR *k* = 1,420, *N* = 336); no post‐death imputation. Subgroup eGFR slope +0.05 (−0.02 to +0.12) stage 3 and +0.03 (−0.04 to +0.10) stage 4 (time × stage *p* = 0.412; not equivalence). Residual survivor bias from informative truncation cannot be excluded.

### 3.3 Changes in Renal Function and Biomarkers

**Figure 2:**
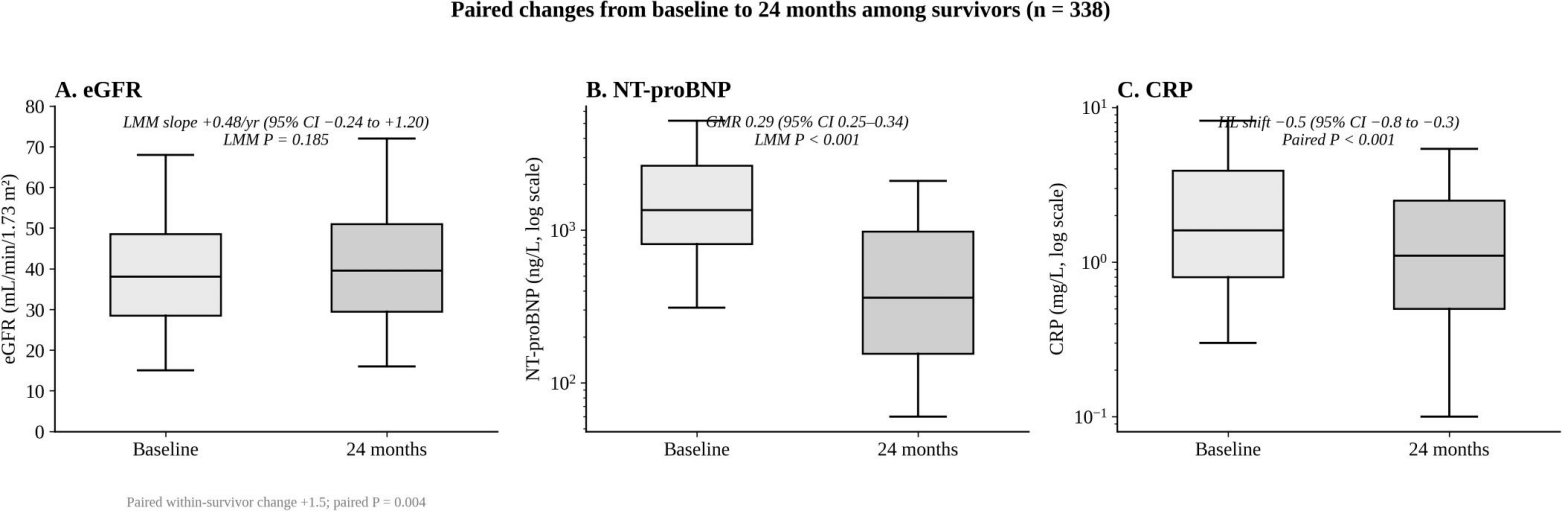
Paired changes in renal function, neurohormonal activation and inflammation from baseline to 24 months among survivors (*n* = 338). Box‐and‐whisker plots show median, interquartile range and whiskers to data extremes. (A) eGFR (mL/min/1.73 m^2^; linear scale): primary emphasis is the total‐cohort (*N* = 360) linear mixed‐model slope (+0.04/month, 95% CI −0.02 to +0.10; annualized +0.48/year, 95% CI −0.24 to +1.20; LMM *p* = 0.185; paired within‐survivor change +1.5, paired *p* = 0.004). (B) NT‐proBNP (ng/L; log scale; geometric mean ratio 0.29, 95% CI 0.25–0.34; LMM *p* < 0.001). (C) CRP (mg/L; log scale; Hodges–Lehmann shift −0.5, 95% CI −0.8 to −0.3; paired *p* < 0.001).

Over 24‐months, mean eGFR remained essentially unchanged (38.6 ± 13.8 to 40.1 ± 14.2 mL/min/1.73 m^2^ in survivors; paired *p* = 0.004; mixed‐model slope +0.04/month, *p* = 0.185) and serum creatinine showed no significant increase (1.71 ± 0.52 vs 1.68 ± 0.50 mg/dL; *p* = 0.038) (Figure 2). Median NT‐proBNP fell from 1350 [810–2650] to 360 [155–980] ng/L (*p* < 0.001) and median CRP from 1.6 [0.8–3.9] to 1.1 [0.5–2.5] mg/L (*p* < 0.001), while haemoglobin, sodium, potassium, albumin and cholesterol remained stable (Table 2).

### 3.4 NYHA Functional Class and Clinical Status

**Figure 3:**
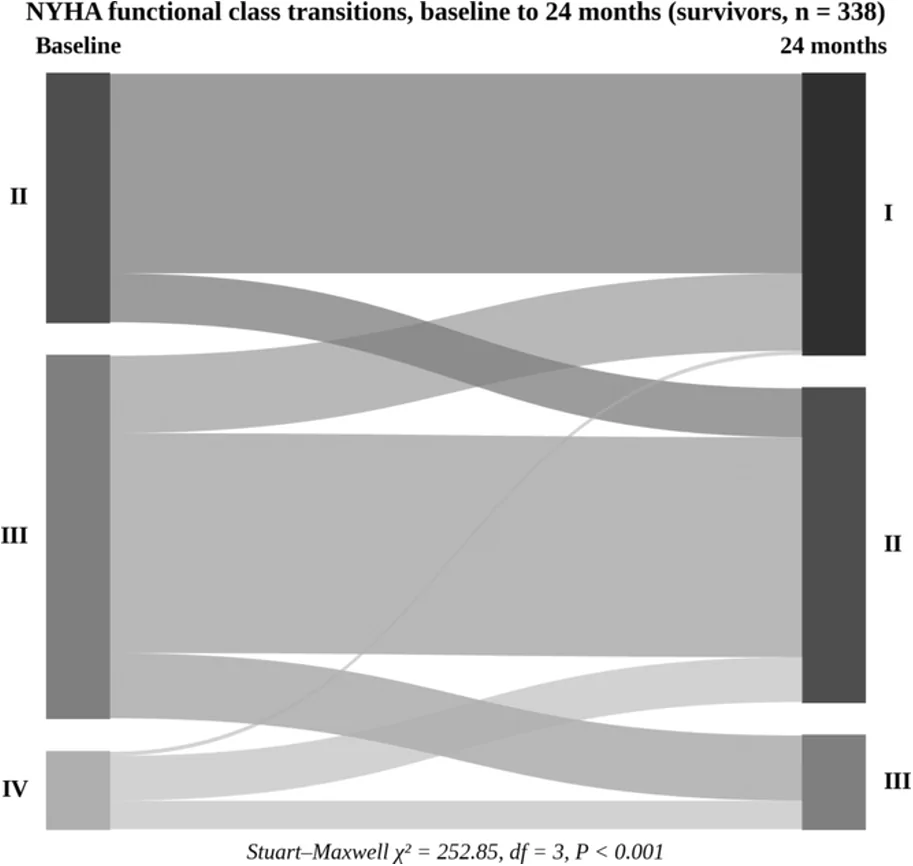
NYHA functional class transitions from baseline to 24 months among survivors (*n* = 338). Stream widths are proportional to patient numbers. No patient was in NYHA class I at baseline; at 24 months 86.4% (292/338) were in class I (40.8%) or II (45.6%) and no survivor remained in class IV (vs 11.2% at baseline). Stuart–Maxwell marginal‐homogeneity test *χ* ^2^ = 252.85, df = 3, *p* < 0.001. NYHA, New York Heart Association.

Functional status improved markedly under sacubitril/valsartan. At baseline no patient was in NYHA class I, whereas at 24%–40.8% were in class I and 45.6% in class II; class III fell from 52.7% to 13.6% and no patient remained in class IV (Table 2). The Sankey diagram (Figure 3) and the transition matrix in Table S2 illustrates the predominant shift from classes III and IV to classes II and I, with little deterioration in functional class. The 6‐min walk distance increased from 322 ± 65 to 388 ± 71 m (*p* < 0.001), and in a responder analysis assigning the worst rank to patients who died, the direction of improvement persisted.

Because eligibility required baseline NYHA class II–IV, no patient was in class I at baseline by design; the shift into class I reflects improvement from a floor set by the inclusion criteria rather than a change in an unrestricted distribution.

### 3.5 Adverse Events and Tolerability

Sacubitril/valsartan was generally well tolerated in this HFrEF and stage 3–4 CKD cohort. During 24‐months, hyperkalaemia occurred in 44 patients (12 percent), symptomatic hypotension in 42 (11 percent), sustained ≥ 30% eGFR decline (chronic, confirmed) in 8 (2 percent) and chronic dialysis or progression to end‐stage kidney disease in 4 (1 percent). Cardiovascular and all‐cause mortality were 3.9% and 6.1%, respectively, and temporary treatment interruption occurred in 22 patients (6 percent) (Table 3). Most events were controlled with dose reduction or brief interruption; permanent discontinuation was rare and occurred exclusively in patients who subsequently died (*n* = 8; Table S4).

**Table 3:** Adverse events and clinical endpoints over 24 months, by CKD stage.

Adverse event/endpoint	Total (*N* = 360)	Stage 3 (*n* = 176)	Stage 4 (*n* = 184)	*p*	Test
Hyperkalemia (K ≥ 5.5 mmol/L)	44 (12.2)	16 (9.1)	28 (15.2)	0.076	Chi‐square
Symptomatic hypotension (SBP < 100 + sx)	42 (11.7)	18 (10.2)	24 (13.0)	0.405	Chi‐square
Temporary S/V interruption	22 (6.1)	8 (4.5)	14 (7.6)	0.225	Chi‐square
Permanent S/V discontinuation	8 (2.2)	1 (0.6)	7 (3.8)	0.068	Fisher
All‐cause mortality	22 (6.1)	7 (4.0)	15 (8.2)	0.101	Log‐rank
Cardiovascular mortality	14 (3.9)	4 (2.3)	10 (5.4)	0.123	Gray

*Note: n* (%) of patients with ≥ 1 event over 24 months. All permanent S/V discontinuations (*n* = 8) occurred exclusively in non‐survivors; no surviving patient discontinued before 24 months. Stage comparisons by Pearson chi‐square/Fisher exact (non‐fatal) or log‐rank (mortality). Non‐CV deaths (*n* = 8) censored in CV‐mortality KM.

Abbreviations: CKD, chronic kidney disease; eGFR, estimated glomerular filtration rate; ESKD, end‐stage kidney disease; S/V, sacubitril/valsartan.

**Table 3.1:** Primary renal composite—hierarchical component breakdown.

Renal composite component (time‐to‐first)	Total (*N* = 360)	Stage 3	Stage 4	Median event time, mo [IQR]	*p* (stage)
Primary renal composite (total)	12 (3.3)	3 (1.7)	9 (4.9)	14.5 [9.2–18.8]	0.088 (Gray)
Sustained ≥ 30% eGFR decline only	8 (2.2)	2 (1.1)	6 (3.3)	13.0 [8.5–16.5]	—
Progression to ESKD	4 (1.1)	1 (0.6)	3 (1.6)	17.5 [14.0–21.0]	—
Maintenance dialysis (HD/PD)	2 (0.6)	0 (0.0)	2 (1.1)	18.0 [15.5–20.5]	—
Sustained eGFR < 15 without dialysis	2 (0.6)	1 (0.6)	1 (0.5)	16.5 [13.0–20.0]	—
Renal death	0 (0.0)	0 (0.0)	0 (0.0)	—	—

*Note:* Hierarchical time‐to‐first classification; each patient counted once, mutually exclusive subsets (8 + 4 = 12). Renal death explicitly reported as *n* = 0. Patients reaching ESKD had already met the ≥ 30% decline criterion, so ESKD added no new composite events. None of the 12 renal‐composite patients died within 24 months; all 22 deaths were competing first events.

Abbreviations: eGFR, estimated glomerular filtration rate; ESKD, end‐stage kidney disease.

### 3.6 Renal Composite Endpoint and Predictors

**Figure 4:**
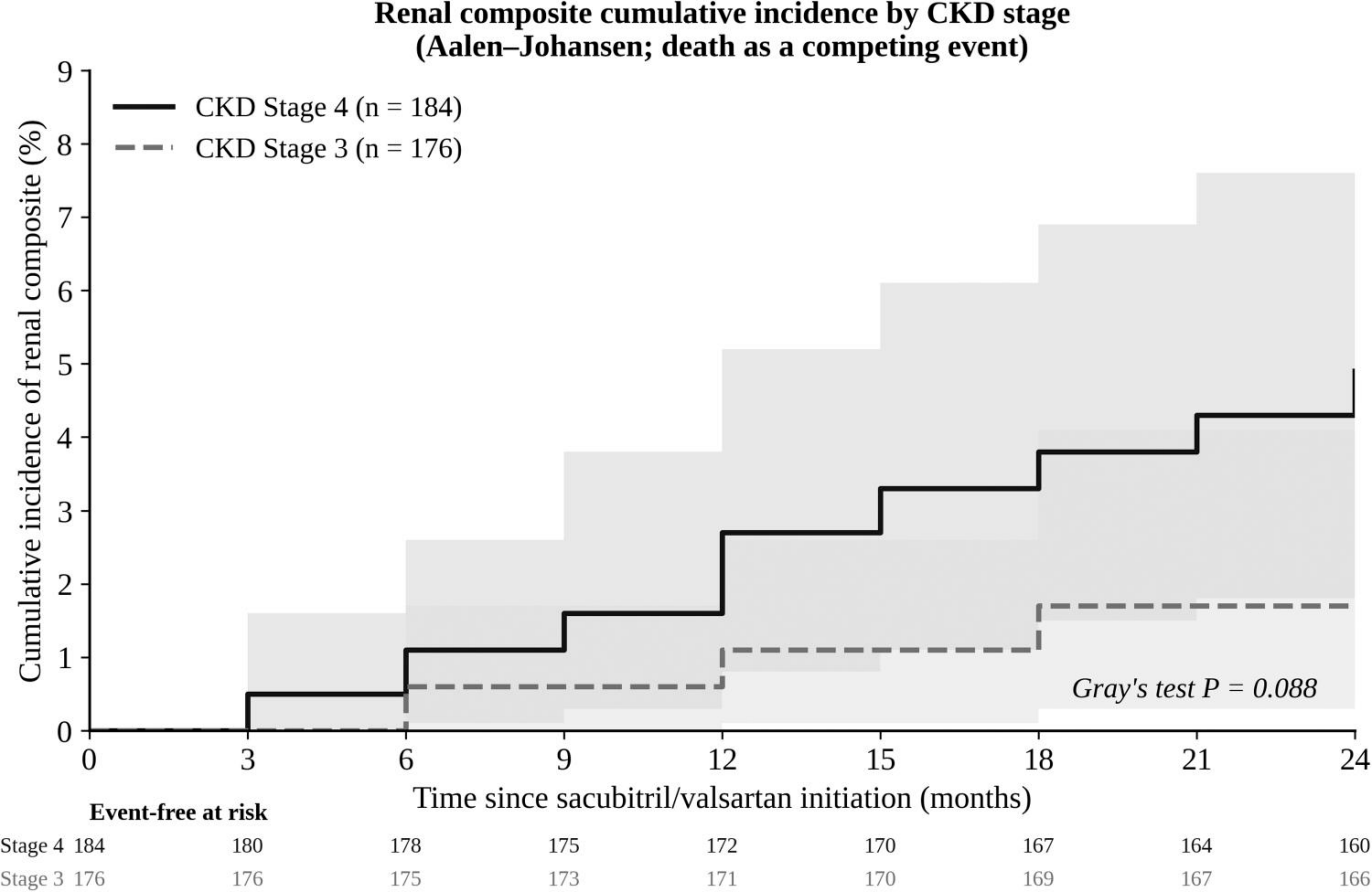
Cumulative incidence of the primary renal composite endpoint by CKD stage. Aalen–Johansen cumulative incidence function (CIF) estimates with 95% confidence intervals (shaded bands), treating all‐cause death (*n* = 22) as a competing event. The event‐free number at risk is shown below the plot. At 24 months the renal composite incidence was 1.7% (95% CI: 0.3–4.1; 3/176) in Stage 3 and 4.9% (95% CI: 2.2–8.4; 9/184) in Stage 4 (Gray's test *χ* ^2^ = 2.91, *p* = 0.088). CKD, chronic kidney disease; eGFR, estimated glomerular filtration rate.

The predefined renal composite endpoint (≥ 30 percent eGFR decline, chronic dialysis or progression to end‐stage kidney disease) was infrequent over 24‐months. The renal composite occurred in 12 patients (3.3%). In Fine–Gray competing‐risk analysis (death as a competing event), lower baseline eGFR (subdistribution HR: 1.45 per 5 mL/min/1.73 m^2^ decrease, 95% CI: 1.10–1.91) and higher baseline UACR (sHR: 1.62 per doubling, 1.15–2.28) were associated with the renal composite, whereas age and diabetes were not (Table 4). Consistentlythe cumulative incidence was higher in stage 4 than in stage 3 CKD (Figure 4; Gray's *p* = 0.088) (Table 3.1) underscoring the prognostic importance of baseline renal function.

**Table 4:** Exploratory associations with the primary renal composite (Fine–Gray + Firth‐penalized Cox).

Variable	Univariable sHR (95% CI)	Univ. *p*	Firth‐penalized HR (95% CI)	Firth *p*
Age (per 10‐year increase)	1.18 (0.84–1.66)	0.334	—	—
Diabetes mellitus (yes vs no)	2.15 (0.68–6.80)	0.192	—	—
Baseline eGFR (per 5 mL/min/1.73 m^2^ decrease)	1.45 (1.10–1.91)	0.008	1.38 (1.04–1.84)	0.024
Baseline UACR (log_2_, per doubling; *n* = 336)	1.62 (1.15–2.28)	0.006	1.51 (1.06–2.15)	0.022

*Note: N* = 360; renal composite events *n* = 12; competing deaths *n* = 22. Univariable = Fine–Gray subdistribution HR (sHR). eGFR and UACR pre‐specified a priori (not data‐driven). To avoid overfitting (EPV = 6.0), the multivariable model was a 2‐predictor Firth‐penalized cause‐specific Cox (complete‐case *N* = 336, 12 events); presented as exploratory sensitivity analysis; stability by 1000 bootstrap resamples.

Abbreviations: CI, confidence interval; eGFR, estimated glomerular filtration rate; EPV, events per variable; HR, hazard ratio; sHR, subdistribution hazard ratio; S/V, sacubitril/valsartan; UACR, urine albumin‐to‐creatinine ratio.

### 3.7 Mortality and Clinical Predictors

**Figure 5:**
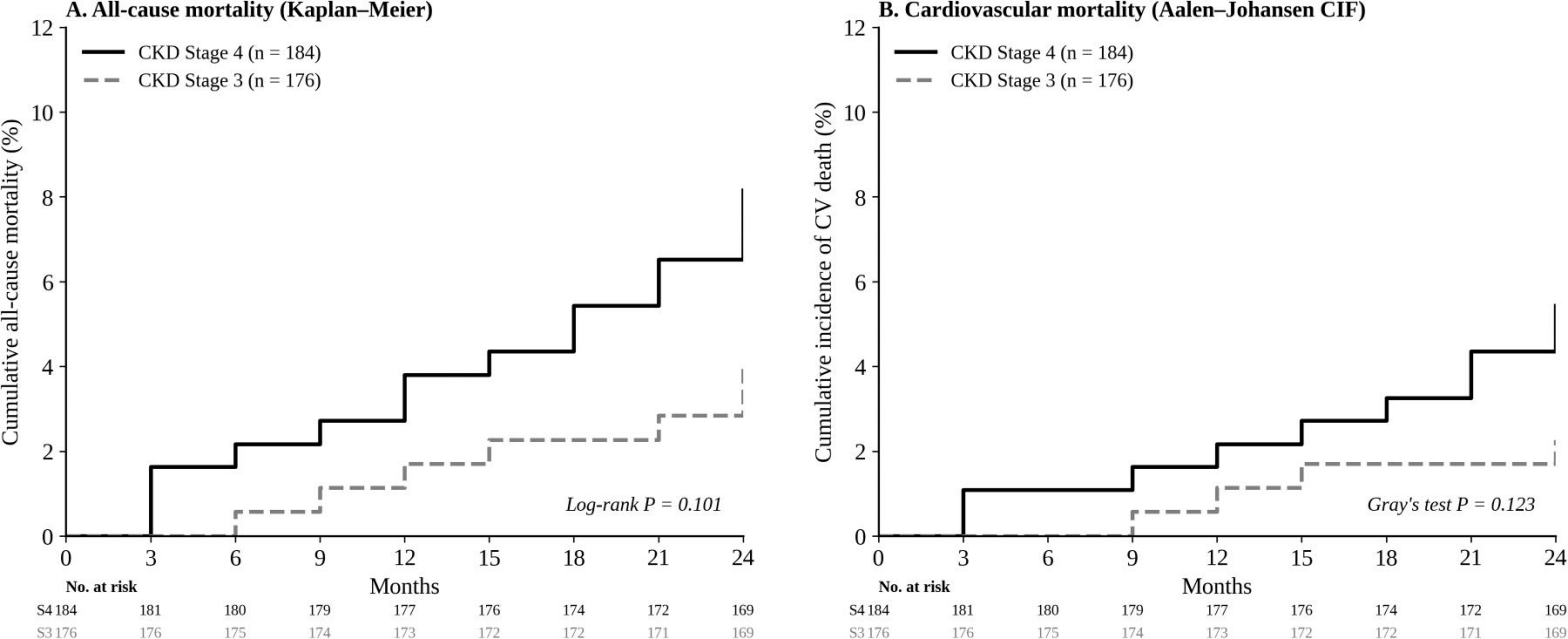
All‐cause and cardiovascular mortality by CKD stage. (A) Kaplan–Meier all‐cause mortality (22 deaths; Stage 3 7/176 [4.0%] vs Stage 4 15/184 [8.2%]; log‐rank *p* = 0.101; 24‐month survival 96.0% vs 91.8%). (B) Aalen–Johansen cumulative incidence of cardiovascular death (14 deaths; Stage 3 4/176 [2.3%] vs Stage 4 10/184 [5.4%]) treating non‐cardiovascular death (*n* = 8) as a competing event (Gray's test *p* = 0.123). Number‐at‐risk arrays are shown below each panel. CKD, chronic kidney disease.

During 24 months, all‐cause and cardiovascular mortality were 6.1% and 3.9%, respectively (Table 3; Figure 5); baseline characteristics of survivors versus non‐survivors are summarized in Table 1b. In a Firth‐penalized Cox model (chosen for the limited number of events), older age, baseline NYHA class III–IV and higher log NT‐proBNP were associated with higher all‐cause mortality, while higher baseline eGFR was borderline protective; bootstrap resampling confirmed the direction of these associations (Table 5).

**Table 5:** Multivariable Firth‐penalized Cox regression for all‐cause mortality.

Predictor	Univariable HR (95% CI)	Univ. *P*	Firth‐penalized multivariable HR (95% CI)	Firth *P*
Age (per 10‐year increase)	1.48 (1.08–2.03)	0.015	1.39 (1.01–1.92)	0.044
NYHA class III–IV (yes vs no)	3.12 (1.21–8.05)	0.018	2.65 (1.02–6.88)	0.046
Baseline eGFR (per 5 mL/min/1.73 m^2^ decrease)	1.28 (1.06–1.55)	0.010	1.22 (1.01–1.48)	0.041
Baseline NT‐proBNP (log_2_, per doubling)	1.54 (1.18–2.01)	0.002	1.41 (1.06–1.88)	0.018

*Note: N* = 360; all‐cause deaths *n* = 22. Predictors pre‐specified a priori (clinical severity/neurohormonal prognosis), not data‐driven. S/V maintenance dose intentionally excluded from baseline Cox to prevent immortal‐time bias (evaluated separately in 3‐month landmark analyzes). EPV = 5.5 (22 events/4 predictors) → Firth‐penalized Cox with profile‐likelihood 95% CI; stability by 1000 bootstrap resamples.

Abbreviations: CI, confidence interval; eGFR, estimated glomerular filtration rate; EPV, events per variable; HR, hazard ratio; NYHA, New York Heart Association; NT‐proBNP, N‐terminal pro–B‐type natriuretic peptide; S/V, sacubitril/valsartan.

Because the dose prescribed at the 3‐month landmark is reached only after survival and tolerance, the dose–mortality relationship was examined by 3‐month landmark analysis and is interpreted as descriptive and confounded by treatment tolerance rather than causal (see below).

### 3.8 Kaplan–Meier Analyzes by Sacubitril/Valsartan Dose, CKD Stage and SGLT2 Use

Kaplan–Meier curves for the composite of all‐cause mortality plus renal events separated by sacubitril/valsartan maintenance dose, with the target dose showing the lowest event rates and the lowest dose the highest. Because achieved dose is attained only after survival and tolerance, the dose–outcome relationship is examined in a 3‐month landmark analysis (Figure S1) and interpreted descriptively. Stratification by CKD stage showed more events in stage 4 than in stage 3 (Figure 4). Exploratory curves by SGLT2 inhibitor use suggested fewer composite events with SGLT2 therapy; after adjustment for calendar era and time‐varying exposure this association was attenuated (Figure S2) and is regarded as hypothesis‐generating. In sensitivity analysis by enrollment period, the direction of the association was similar, although statistical certainty was reduced.

In a 3‐month landmark analysis stratified by the dose prescribed at the 3‐month landmark, all‐cause mortality was numerically higher with the low dose (7.9%) than the intermediate (3.2%) or target (2.8%) dose (Figure S1; log‐rank *p* = 0.042); landmark cohort definitions and pre‐event counts are given in Table S5; however, all permanent discontinuations occurred in patients who subsequently died, consistent with confounding by treatment tolerance and possible reverse causation rather than a protective effect of higher dosing. The renal composite cumulative incidence did not differ across dose strata (Gray's *p* = 0.418), with no statistically discernible between‐dose difference, although these estimates were imprecise. Concomitant SGLT2‐inhibitor use, modeled as a time‐varying exposure adjusted for calendar era, was associated with lower all‐cause mortality (adjusted HR: 0.61, 95% CI: 0.37–0.98) and a non‐significant trend for the renal composite (adjusted HR: 0.68, 0.35–1.32) (Figure S2); these SGLT2‐inhibitor analyzes are exploratory and hypothesis‐generating.

## 4 Discussion

In this single‐centre real‐world cohort of 360 patients with HFrEF and stage 3–4 CKD, 24‐month sacubitril/valsartan therapy was associated with a stable mean renal trajectory and low rates of severe kidney outcomes. NT‐proBNP and CRP fell substantially, NYHA class improved, and most adverse events such as hyperkalaemia, symptomatic hypotension and renal worsening were manageable, with few permanent discontinuations. Between‐dose and SGLT2‐inhibitor comparisons were exploratory and imprecise and are not interpreted as evidence of benefit. Overall, sacubitril/valsartan was associated with a stable mean renal trajectory in this high‐risk population; comparative renal outcomes and tolerability and any dose effect cannot be established from an uncontrolled, single‐arm design. These findings are particularly relevant in routine clinical practice, where patients with advanced CKD are often excluded from randomized trials and clinicians remain cautious about initiating ARNI therapy.

Our results suggest that sacubitril/valsartan has a stable mean eGFR trajectory in HFrEF patients with advanced CKD. Mean eGFR remained essentially unchanged around 40–41 mL/min/1.73 m^2^ over 24‐months, dialysis or progression to end‐stage kidney disease occurred in ~1%, and renal composite events were infrequent. In exploratory competing‐risk analysis, lower baseline eGFR and higher UACR were associated with the renal composite; these associations are hypothesis‐generating. These findings align with PARADIGM HF/PARAGON HF and real‐world data in predominantly stage 1–3 CKD, where eGFR decline on sacubitril/valsartan is slower than with ACEIs/ARBs [2, 13]. By focusing on CKD stages 3–4 with 24‐month follow‐up, our study adds hypothesis‐generating evidence that ARNI therapy can be compatible with a stable renal trajectory in this high‐risk group under close monitoring.

In our cohort, sacubitril/valsartan therapy was associated with a marked and sustained reduction in both NT‐proBNP and CRP levels over 24‐months. The pronounced decline in NT‐proBNP is consistent with prior reports in HFrEF populations, but most existing data derive from patients with preserved or only mildly to moderately impaired renal function and from relatively short follow‐up periods [14, 15]. The parallel and statistically significant decrease in CRP observed in our study may suggest a concomitant attenuation of systemic inflammatory burden, which has been less frequently evaluated, particularly in advanced CKD [16]. Taken together, these findings may be compatible with the combined hemodynamic, neurohormonal, and potentially anti‐inflammatory effects of sacubitril/valsartan, and they provide additional, hypothesis‐generating evidence supporting its use in high‐risk HFrEF patients with stage 3–4 CKD [17].

Similarly, NYHA functional class improved markedly during follow‐up. While no patient was in class I at baseline, by 24‐months about 40% were in class I and 46% in class II, with very few remaining in classes III–IV and none in class IV. This sustained shift toward lower NYHA class reflects a change in symptom class and functional capacity beyond the 6–12 months typically reported; no validated quality‐of‐life instrument was used [18, 19]. When compared with existing ARNI series in CKD and with general HFrEF cohorts, our 24‐month rates of 86% in NYHA I–II appear substantially higher, resembling the more dramatic functional improvement reported only in small refractory HF cohorts undergoing intensive interventions such as dialysis or peritoneal dialysis [20, 21]. The concurrent decrease in loop diuretic use (76% to 62% over 24‐months) further supports a net improvement in clinical stability and congestion, although changes in diuretic prescribing may also reflect overall optimization of guideline directed therapy.

The magnitude of functional improvement should be interpreted in light of the management model applied in our centre, which mirrors the intensive strategy tested in STRONG‐HF: early quadruple guideline‐directed therapy, early and structured dose optimization, and close follow‐up with symptom, examination and volume assessment at each visit. Rather than unexpected, the favorable functional trajectory is consistent with rapid protocolised optimization and was corroborated by objective 6‐min walk testing. Nonetheless, NYHA class remains subjective and, in this retrospective design, susceptible to documentation and ascertainment bias, and no validated quality‐of‐life instrument (KCCQ or MLHFQ) was administered; the results reflect functional capacity rather than quality of life and should be interpreted with caution [22].

In our selected cohort, sacubitril/valsartan was generally well tolerated despite the high‐risk profile of stage 3–4 CKD. Rates of hyperkalaemia (12%) and symptomatic hypotension (11%) were within the range reported in comparable CKD–HF series and were usually managed with dose adjustment or temporary interruption; clinically relevant worsening of renal function occurred in only 2% of patients [23, 24]. Chronic dialysis or progression to end‐stage kidney disease (~1%), all‐cause mortality (6.1%) and cardiovascular mortality (3.9%) were low over 24‐months, and permanent discontinuation was rare. The observed tolerability rates were within previously reported ranges; however, comparative safety cannot be established in this uncontrolled cohort [16, 25].

Concomitant SGLT2 inhibitor use rose markedly after 2021, reflecting rapid uptake of contemporary quadruple therapy in HFrEF with CKD. In this setting, Kaplan–Meier curves suggested fewer composite events in patients receiving SGLT2 inhibitors in addition to sacubitril/valsartan, in line with emerging data and meta‐analytic signals indicating additive benefits of combining ARNI and SGLT2i [26, 27]. However, this exploratory, observational analysis is prone to confounding by indication and temporal improvements in care, so any causal interpretation should remain cautious [28]. This signal should be viewed as hypothesis‐generating. Calendar time and treatment selection may have influenced the observed difference.

Taken together, our findings suggest that sacubitril/valsartan may be a reasonable option even in HFrEF patients with stage 3–4 CKD, a group in whom ARNI is often avoided [29]. In this high‐risk population, renal function was largely preserved over 24‐months, NT‐proBNP and CRP fell substantially, NYHA class improved, and rates of death and severe renal events were relatively low. Because the design is observational, single‐arm and uncontrolled, these observations should be regarded as hypothesis‐generating rather than as a treatment recommendation [30, 31]. Importantly, patients with advanced CKD are frequently underrepresented in major heart failure trials, making real‐world evidence particularly valuable for this vulnerable population.

Our study has several strengths. It includes a relatively large, well‐defined cohort of 360 patients with HFrEF and stage 3–4 CKD, a clearly defined high‐risk group, and provides 24‐months of follow‐up, longer than many real‐world reports, allowing assessment of long‐term renal and clinical outcomes [5]. The analysis was performed in a contemporary treatment era with substantial SGLT2 inhibitor use and current ESC/KDIGO‐guided care, and examined a wide range of endpoints, including a detailed renal composite (≥ 30% eGFR decline, dialysis, or progression to end‐stage kidney disease), all‐cause and cardiovascular mortality, maintenance dose categories, and simple clinical predictors (age, baseline eGFR, NYHA class, NT‐proBNP) [32]. Finally, the real‐world design with minimal exclusions yields a study population that closely mirrors daily practice rather than the highly selected cohorts of randomized trials [33].

The limitations of this study should be acknowledged. First, its retrospective, single‐centre observational design and the absence of a control group prevent causal inference and direct comparison with ACEI/ARB therapy. As a real‐world cohort, a degree of selection bias cannot be entirely excluded. However, the study population reflects routine clinical practice and includes patients with advanced chronic kidney disease (stage 3–4), a group that is frequently underrepresented in randomized clinical trials. Second, some biomarkers and imaging parameters, such as longitudinal eGFR slope, proteinuria, or detailed cardiac remodeling indices, were not systematically available at standardized intervals. Third, although we explored the potential impact of sacubitril/valsartan dose and concomitant SGLT2 inhibitor therapy, these analyzes remain observational and may be influenced by residual confounding or treatment selection bias. Finally, dialysis patients and those with stage 5 CKD were excluded, so the findings cannot be generalized to end‐stage kidney disease. Despite these limitations, the relatively large cohort, the focus on stage 3–4 CKD, and the 24‐month follow‐up provide meaningful real‐world insights into the renal safety and clinical outcomes of sacubitril/valsartan in this high‐risk population.

Only 10 of 370 eligible patients (2.7%) were excluded for unretrievable follow‐up, reflecting administrative or geographic factors (data held abroad, non‐adherence, and one heart transplantation at month 3, a post‐baseline event); the national e‐Nabız record further limited in‐country loss to follow‐up. Because adequate follow‐up data were unavailable for the excluded patients, their clinical comparability with the included cohort could not be formally assessed, and residual selection bias therefore cannot be excluded. One excluded expatriate patient had records only through month 6 and was later reported by family to have died abroad approximately 3 years after enrollment—well beyond the 24‐month window—so this death was not an endpoint. Nonetheless, paired 24‐month comparisons are inherently restricted to survivors, and residual selection and survivor effects cannot be fully excluded; accordingly, findings are interpreted as descriptive and hypothesis‐generating rather than as evidence of comparative efficacy or renal outcomes and tolerability.

In conclusion, in patients with HFrEF and stage 3–4 CKD treated with S/V, 24‐month follow‐up was associated with stable renal function, reduced symptom burden and relatively low rates of death and severe renal events. Any apparent dose–outcome gradient is likely confounded by treatment tolerance (reverse causation) and is not interpreted causally. These descriptive findings are hypothesis‐generating and require confirmation in larger prospective controlled studies.

## Author Contributions

S.D., F.A., E.T. and O.O.Ş. contributed to study conception, data collection, analysis, and drafting. M.B.Y. served as Senior Mentor, providing input in study design and critical revision of the manuscript.

## Funding

The authors have nothing to report.

## Ethics Statement

The study protocol was approved by the Clinical Research Ethics Committee of Ordu University (decision no. 2026/165). The study was conducted in accordance with the Declaration of Helsinki. Patient data were de‐identified before analysis.

## Consent

Owing to the retrospective design, the requirement for written informed consent was waived by the Clinical Research Ethics Committee of Ordu University (decision no. 2026/165).

## Conflicts of Interest

The authors declare no conflicts of interest.

## Supporting information


**Figure S1:** Three‐month landmark analysis by sacubitril/valsartan dose prescribed at the landmark. Outcomes are evaluated from the month‐3 landmark (post‐landmark months 0–21 = study months 3–24) to remove immortal‐time bias; patients are classified by the dose prescribed at the 3‐month landmark (Low 24/26, Intermediate 49/51, Target 97/103 mg bid). (A) Kaplan–Meier all‐cause mortality (landmark cohort n = 357; low 7.9%, intermediate 3.2%, target 2.8%; log‐rank P = 0.042). (B) Aalen–Johansen renal composite CIF (landmark cohort n = 356; low 4.3%, intermediate 1.9%, target 2.8%; Gray's test P = 0.418). These analyses are exploratory. bid, twice daily; CIF, cumulative incidence function.

**Figure S2:** Forest plot of exploratory naive and time‐dependent adjusted hazard ratios for concomitant SGLT2‐inhibitor therapy. SGLT2‐inhibitor exposure was modelled as a time‐varying covariate adjusted for calendar era (before vs from 2021), baseline eGFR and diabetes mellitus (N = 360; 697.5 person‐years). Squares/diamonds are hazard ratios; bars are 95% confidence intervals. All‐cause mortality: naive HR 0.43 (0.19–0.96), adjusted HR 0.61 (0.37–0.98, P = 0.041). Renal composite: naive HR 0.44 (0.14–1.38), adjusted HR 0.68 (0.35–1.32, P = 0.254). These exploratory, hypothesis‐generating estimates are imprecise given only 22 deaths and 12 renal events. HR, hazard ratio; CI, confidence interval; SGLT2i, sodium‐glucose cotransporter‐2 inhibitor.

Supporting File

## Data Availability

The data that support the findings of this study are available on request from the corresponding author. The data are not publicly available due to privacy or ethical restrictions.
