# Supplementary material for: Long‐Term Renal Function and Clinical Outcomes of Sacubitril/Valsartan in HFrEF and Stage 3–4 Chronic Kidney Disease: A 24‐Month Single‐Centre Cohort
Source: Clin Cardiol. 2026 Sep 23;49(9):e70473. doi: 10.1002/clc.70473 (PMC13599594; doi:10.1002/clc.70473)
Supplement: Supplementary file 1 [file CLC-49-e70473-s002.docx]

**Supplementary Tables**

## **Supplementary Table S1. Guideline-directed medical therapy transitions, baseline to 24 months (survivors, n=338)**

| **GDMT class** | **Continued** | **Initiated** | **Discontinued** | **Never used** | **Net change** | **McNemar P** |
| --- | --- | --- | --- | --- | --- | --- |
| MRA | 196 (58.0) | 50 (14.8) | 8 (2.4) | 84 (24.8) | +12.4 pp | < 0.001 |
| SGLT2 inhibitor | 116 (34.3) | 158 (46.7) | 4 (1.2) | 60 (17.8) | +45.6 pp | < 0.001 |
| Beta-blocker | 280 (82.8) | 11 (3.3) | 5 (1.5) | 42 (12.4) | +1.8 pp | 0.210 |
| Loop diuretic | 198 (58.6) | 10 (3.0) | 56 (16.6) | 74 (21.9) | −13.6 pp | < 0.001 |
| Four-pillar GDMT | 78 (23.1) | 164 (48.5) | 2 (0.6) | 94 (27.8) | +47.9 pp | < 0.001 |

*Surviving patients, n (% of n=338). Net change = initiated − discontinued (percentage points). Exact McNemar test.* **Abbreviations:** GDMT, guideline-directed medical therapy; ACEi, angiotensin-converting enzyme inhibitor; ARB, angiotensin receptor blocker; MRA, mineralocorticoid receptor antagonist; SGLT2i, sodium-glucose cotransporter-2 inhibitor; S/V, sacubitril/valsartan.

## **Supplementary Table S2. NYHA functional class transition matrix, baseline to 24 months (survivors, n=338)**

| **Baseline (T0)** | **24-mo NYHA I** | **II** | **III** | **IV** | **Total baseline** |
| --- | --- | --- | --- | --- | --- |
| NYHA I | 0 (0.0) | 0 (0.0) | 0 (0.0) | 0 (0.0) | 0 (0.0) |
| NYHA II | 98 (29.0) | 24 (7.1) | 0 (0.0) | 0 (0.0) | 122 (36.1) |
| NYHA III | 38 (11.2) | 108 (32.0) | 32 (9.5) | 0 (0.0) | 178 (52.7) |
| NYHA IV | 2 (0.6) | 22 (6.5) | 14 (4.1) | 0 (0.0) | 38 (11.2) |
| **Total 24-mo** | 138 (40.8) | 154 (45.6) | 46 (13.6) | 0 (0.0) | 338 (100) |

*Surviving patients, n (% of n=338). Stuart–Maxwell marginal homogeneity χ²=252.85, df=3, P<0.001.* **Abbreviations:** NYHA, New York Heart Association.

## **Supplementary Table S3. Sacubitril/valsartan starting-to-maintenance dose transition matrix (survivors, n=338)**

| **Baseline starting dose** | **24-mo Low** | **Intermediate** | **Target** | **Total** |
| --- | --- | --- | --- | --- |
| 24/26 mg bid (Low) | 62 (18.3) | 71 (21.0) | 20 (5.9) | 153 (45.3) |
| 49/51 mg bid (Intermediate) | 14 (4.1) | 77 (22.8) | 59 (17.5) | 150 (44.4) |
| 97/103 mg bid (Target) | 2 (0.6) | 12 (3.6) | 21 (6.2) | 35 (10.4) |
| **Total 24-mo** | 78 (23.1) | 160 (47.3) | 100 (29.6) | 338 (100) |

*Surviving patients, n (% of n=338). Stuart–Maxwell χ²=80.35, df=2, P<0.001. Among patients starting at 24/26 mg bid, 59.5% (91/153) up-titrated to intermediate/target dose by 24 months.* **Abbreviations:** S/V, sacubitril/valsartan; bid, twice daily.

## **Supplementary Table S4. Timing and reasons for permanent sacubitril/valsartan discontinuation among non-survivors (n=8)**

| Case / stage | **Discontinuation** | **Primary reason** | **Death** | **Vs 3-mo landmark** |
| --- | --- | --- | --- | --- |
| Case 1 (St4) | Mo 1.4 | Refractory hypotension / cardiogenic shock | Mo 1.8 | Before |
| Case 2 (St4) | Mo 2.1 | Terminal deterioration / low-output HF | Mo 2.5 | Before |
| Case 3 (St4) | Mo 2.6 | Refractory hyperkalemia (K=6.1) | Mo 2.9 | Before |
| Case 4 (St4) | Mo 4.8 | Severe progressive hypotension (SBP<85) | Mo 5.4 | After |
| Case 5 (St4) | Mo 6.5 | Acute-on-chronic renal worsening / collapse | Mo 7.2 | After |
| Case 6 (St3) | Mo 10.8 | Terminal HF decompensation / palliative | Mo 11.5 | After |
| Case 7 (St4) | Mo 12.1 | Refractory hyperkalemia / renal progression | Mo 13.4 | After |
| Case 8 (St4) | Mo 14.2 | Hemodynamic instability / severe orthostasis | Mo 15.6 | After |

*All 8 permanent discontinuations occurred in patients who subsequently died (3 before, 5 after the 3-month landmark; median post-landmark 10.8 mo). Discontinuation and lower achieved dose were driven by intolerance and terminal severity, supporting reverse causation in observational dose–mortality associations.* **Abbreviations:** S/V, sacubitril/valsartan.

## **Supplementary Table S5. Three-month landmark pre-event counts and cohort definitions**

| **Landmark parameter** | **Total** | **Stage 3** | **Stage 4** | **Notes** |
| --- | --- | --- | --- | --- |
| All-cause deaths before month 3 | 3 (0.8) | 0 | 3 (1.6) | Event months 1.8, 2.5, 2.9 (Stage 4) |
| Renal composite events before month 3 | 1 (0.3) | 0 | 1 (0.5) | First qualifying at mo 2.4 (confirmed 5.6) |
| Mortality landmark cohort | 357 | 176 | 181 | Evaluated month 3–24 |
| Renal event-free landmark cohort | 356 | 176 | 180 | Evaluated month 3–24 |
| Composite event-free landmark cohort | 356 | 176 | 180 | Evaluated month 3–24 |

*3-month landmark (median time to dose optimization 2.2 mo, IQR 1.4–3.1) prevents immortal-time/look-ahead bias. Dose classified by dose at month 3, without reference to future trajectory.* **Abbreviations:** S/V, sacubitril/valsartan.
